# Clinical Trials in Pediatric Autosomal Dominant Polycystic Kidney Disease

**DOI:** 10.3389/fped.2017.00053

**Published:** 2017-03-23

**Authors:** Melissa A. Cadnapaphornchai

**Affiliations:** ^1^University of Colorado Anschutz Medical Campus, Aurora, CO, USA

**Keywords:** autosomal dominant polycystic kidney disease, child, pediatric, clinical trials, therapy

## Abstract

Autosomal dominant polycystic kidney disease (ADPKD) is the most common hereditary kidney disease and is associated with concerning long-term implications for kidney function and cardiovascular health. Early intervention is needed in order to mitigate these long-term complications. Herein, we review important findings from recent clinical trials in ADPKD and their relevance to affected children and young adults and consider future directions for intervention. Recent clinical trials support aggressive control of blood pressure with blockade of the renin-angiotensin-aldosterone system as well as potential benefit of pravastatin therapy in children and young adults with ADPKD. There are several other candidate therapies, some of which have shown benefit in adult ADPKD, which require further investigation in affected children.

Autosomal dominant polycystic kidney disease (ADPKD) is the most common hereditary kidney disease, affecting one in 500 individuals and accounting for nearly 5% of end-stage kidney disease in the United States (1). Although historically considered a disease of adults, it has become clear in recent decades that ADPKD can have significant clinical manifestations as early as *in utero*. Early intervention to mitigate progression of structural kidney disease is anticipated to have the most long-term impact on outcome. Thus, recent clinical trials have focused on early stage ADPKD and specifically on the potential benefits of intervention during childhood. The purpose of this article is to review important findings from recent clinical trials in children and young adults with ADPKD and to discuss future directions for therapeutic intervention.

## Lessons Learned from Natural History Studies in Children

Observational studies in the 1980s and 1990s in children at risk for ADPKD (i.e., one parent known to have ADPKD) began to delineate the extent of disease in children and to identify specific risk factors for subsequent intervention (2, 3). Through such studies, we learned that children with ADPKD had larger kidneys which grew faster than those of unaffected children. Moreover, the larger the kidneys were at baseline imaging, the faster they grew over time. Ultrasonography was shown to be a reliable non-invasive tool for the diagnosis of ADPKD in children. Specifically with the degree of resolution available in the 1990s, this method could delineate kidney cysts in nearly 80% of children aged 5–18 years with known *PKD1* mutations (2). The presence of bilateral cysts in at-risk children is sufficient for diagnosis (4). Many of the same clinical manifestations, which had typified adult ADPKD, including pain, gross and/or microhematuria, proteinuria, and impaired urinary concentrating ability, were also present in affected children, with frequency of symptoms correlating to extent of structural kidney disease. Early cardiovascular disease was also of concern in the pediatric population, with a high prevalence of hypertension and elevated left ventricular mass index (LVMI) as compared to unaffected children (5, 6). Distinct from adults, however, the vast majority of children with ADPKD maintained normal kidney function despite progressive structural kidney disease, thus emphasizing the great importance of total kidney volume (TKV) as a measureable outcome of early disease progression and the primary focus of therapeutic intervention in the pediatric ADPKD population. Although there is good correlation between ultrasound and magnetic resonance imaging (MRI) assessment of TKV in ADPKD children (7), MRI is the preferred method to follow TKV in the research setting and clearly is a more accurate and reliable measure as structural disease advances (8).

## Blood Pressure Control in ADPKD

Blood pressure control is a critical aspect of chronic kidney disease (CKD) management in both children and adults, with numerous studies showing delayed deterioration in kidney function with more aggressive control (9–11). The prevalence of hypertension in pediatric ADPKD is estimated to be 20% (12), with an even higher prevalence in very early onset ADPKD (13, 14). Previous studies in adults with ADPKD implicated a major role for the renin-angiotensin-aldosterone system (RAAS) in the pathogenesis of hypertension and left ventricular hypertrophy [reviewed in Ref. (15, 16)]. Although postulated, there has however been no direct evidence that RAAS induces kidney cyst growth in this condition.

With these considerations, an interventional trial was designed to assess the effect of aggressive control of blood pressure with angiotensin-converting enzyme inhibition (ACEI) on kidney and cardiovascular disease progression in children and young adults ages 4–21 years with ADPKD (17). Study participants with hypertension (blood pressure above the 95th percentile for height, sex, and age) were randomized to enalapril treatment with goal blood pressure at the 90th percentile (HBP90) or at the 50th percentile (HBP50), while participants with high normal blood pressure (75th–95th percentile for height, sex, and age) were randomized to either enalapril with goal blood pressure at the 50th percentile (NBP50) or to observation without treatment (NBP90). All participants were followed annually for 5 years with routine laboratory testing, 24-h urine creatinine clearance as an estimate of glomerular filtration rate (eGFR), abdominal ultrasound and MRI assessment of TKV, and echocardiographic assessment of LVMI.

Not surprisingly, participants in the HBP50 group required more antihypertensive medications (mean ± SEM: 2.8 ± 0.3 vs. 1.6 ± 0.4; *P* < 0.05) than HBP90 participants. Regardless of the degree of hypertension control, however, participants in both of these study groups demonstrated a significant increase in TKV and decrease in eGFR over the study period. No significant differences were observed in LVMI or urine albumin excretion between study groups or over time. These results emphasized the high risk for structural and functional kidney disease progression associated with hypertension in children with ADPKD.

The clinical trial further demonstrated that baseline LVMI in high normal blood pressure participants was similar to that of hypertensive participants and was elevated compared to those with blood pressure below the 75th percentile, suggesting that early cardiovascular involvement is present even with blood pressure in the upper quartile of the normal range. Both NBP50 and NBP90 groups demonstrated a rise in TKV over time despite BP control with ACEI in the NBP50 group. However, participants in the NBP90 group demonstrated significant decrease in eGFR and increase in LVMI over the study period, while these parameters were stable in the NBP50 group. No significant difference was observed in urine albumin excretion between these study groups at baseline or over time. These findings suggested that children and young adults with ADPKD and high normal blood pressure may represent a particular subgroup for whom rigorous control of blood pressure with ACEI could confer renal functional and cardiovascular protection.

Limitations of this single-center pediatric research study include the relatively small number of participants as compared to adult trials despite nationwide recruitment, the restricted number of participants consistently achieving goal BP in the hypertensive group (40% in HBP50 vs. 70% in HBP90 while 100% of NBP participants met BP goal), and the generous dropout rate (27% overall). The latter speaks to a common struggle associated with longitudinal clinical trials in the pediatric population (18, 19).

Subsequently, the multicenter HALT-PKD study was conducted (20). This double-blind, placebo-controlled trial included 558 hypertensive participants 15–49 years of age with early stage ADPKD (eGFR greater than 60 mL/min/1.73 m^2^) who were randomized to either a standard blood pressure target (120/70–130/80 mm Hg) or a low blood pressure target (95/60–110/75 mm Hg) and to either the ACEI lisinopril plus an angiotensin-receptor blocker (telmisartan) or to lisinopril plus placebo. Serial follow-up was performed for 5–8 years with a primary outcome of annual percentage change in TKV by MRI. The trial included a very limited number of participants in the pediatric age group, with mean age of study participants overall of 36 ± 8 years. Combined lisinopril/telmisartan did not significantly alter the rate of increase in TKV as compared to lisinopril/placebo. However, as compared with standard blood pressure control, rigorous blood pressure control was associated with a small (1.1% per year) but significant reduction in TKV and no change in eGFR. Not surprisingly, there was a 16% increase in dizziness and light headedness in the low blood pressure target group.

It is important to note that in the HALT-PKD study, there was no control arm without RAAS blockade. Thus the ability to assess the specific effect of RAAS blockade on TKV was limited. In addition, there were more participants in the low BP group with *PKD2* mutations (19.8 vs. 13.1%); *PKD2* is associated with less progressive kidney disease as compared to *PKD1* (21). Finally, the percentage of subjects consistently meeting BP goal was limited. Specifically, the systolic and diastolic blood pressures, as measured at home, were on target across all study visits in 40–66 and 58–75% of participants in the low-blood pressure group, respectively, and in 32–48 and 33–52% of those in the standard-blood pressure group, respectively. These factors could each have affected the outcome of the clinical trial.

The KDIGO Controversy Conference on ADPKD has suggested a target blood pressure of less than 140/90 in adults with ADPKD with a stricter goal below 130/80 if macroalbuminuria is present (22) and further recommended management of hypertension in ADPKD children per routine guidelines for the general pediatric population (goal below 90th percentile for age, sex, and height) (23) with RAAS blockade as the preferred first-line treatment for hypertension in ADPKD (22). Despite these recommendations, the above-described clinical trials intimate that more aggressive control of BP might be of value for both kidney and cardiovascular reasons in select ADPKD patients. Further investigations are needed to better characterize the long-term relevance of these observations.

Hyperkalemia and reduced GFR are unusual in pediatric ADPKD as kidney function is usually normal. However, the risk of fetal birth defects with RAAS blockade during pregnancy remains an important topic for discussion when providing such treatment to females of appropriate pubertal development.

## Statin Therapy in Pediatric ADPKD

HMG-CoA reductase inhibitors (statins) have been shown to enhance renal blood flow and GFR and to attenuate inflammation through vascular and glomerular nitric oxide production [reviewed in Ref. (24, 25)]. In an animal model of ADPKD, lovastatin reduced the severity of structural and functional kidney disease (26), and in a small study of ADPKD adults, short-term (4-week) treatment with simvastatin was associated with improved renal blood flow and GFR (27). Although the underlying mechanisms are not well understood, it has been proposed that these renoprotective effects are mediated by statin-related inhibition of G proteins, leading to decreased cell proliferation.

In this context, a 3-year randomized double-blind placebo-controlled phase III clinical trial of pravastatin treatment on kidney and cardiovascular disease progression in children and young adults ages 8–22 years with ADPKD and normal kidney function was conducted (28). The primary outcome variable was a combined endpoint of ≥20% increase in TKV corrected for height (HtTKV), LVMI, or urine albumin excretion over the study period. All participants received ACEI treatment. Fewer participants receiving pravastatin achieved the primary endpoint compared with placebo (69 vs. 88%; *P* = 0.03) (29). This was due primarily to a lower proportion reaching the increase in HtTKV (46 vs. 68%; *P* = 0.03), with similar findings observed between study groups for LVMI (25 vs. 38%; *P* = 0.18) and urine albumin excretion (47 vs. 39%; *P* = 0.50). The percent change in HtTKV adjusted for age, sex, and hypertension status over the 3-year period was significantly decreased with pravastatin (23 ± 3% vs. 31 ± 3%; *P* = 0.02). In mixed-model longitudinal data analysis, the increase in percent growth over 3 years was significantly different between the statin and placebo groups (*P* = 0.01). The medication was well tolerated with no adverse effects on serum liver or muscle enzymes; no participant discontinued pravastatin due to side effects.

The mechanisms of statin effect on kidney cyst growth remain to be fully elucidated. Mass spectrometry-based analysis of biomarkers of endothelial dysfunction, inflammation, and oxidative stress was pursued in this cohort (30). Pravastatin therapy was associated with reduced plasma concentrations of cyclooxygenase- and lipoxygenase-derived plasma lipid mediators (e.g., 9-hydroxyoctadecadienoic acid, 13-hydroxyoctadecadienoic acid, and 15-hydroxyeicosatetraenoic acid) over the course of the research study as compared to placebo. These metabolites have been previously shown to enhance the profibrotic effects of angiotensin II (31) and to activate the peroxisome proliferator-activated receptor γ, which has been implicated in cyst growth (32). Thus reduction *via* statin therapy would be anticipated to be of benefit in ADPKD.

These findings support the use of pravastatin to ameliorate progressive structural kidney disease in children and young adults with ADPKD. Whether statin therapy may be beneficial in earlier childhood (i.e., prior to 8 years of age) or in more advanced ADPKD as in older adults is not currently known and requires study. With a readily available, well-tolerated medication which can mitigate progression of kidney disease in children with ADPKD, however, issues now arise regarding whether/when to screen at-risk children. A thoughtful discussion with families regarding the potential benefits and risks of diagnosis (including potential impact on future life/disability insurance) and implications of treatment on long-term prognosis is necessary. Early intervention remains critical in order to have the most long-term impact on kidney and cardiovascular outcomes (33–35). As with ACEI, potential risks to the fetus with statin exposure during pregnancy should be reviewed as appropriate prior to treatment.

## Clinical Trials in Adults with ADPKD and Implications for Pediatric ADPKD

Results from other recent clinical trials in adults with ADPKD must also be considered, recognizing as always that findings from adult studies are not necessarily generalizable to the pediatric population. In 1989, Grantham et al. reported that in human kidney ADPKD cells (but not normal kidney cells), vasopressin-induced cyclic 3′,5′-adenosine monophosphate (cAMP) stimulated chloride-driven fluid secretion, and B-Raf/Mek/extracellular signal-regulated pathway cell proliferation and dedifferentiation, implicating cAMP as an important regulator of growth in ADPKD kidney cysts (36, 37). Some years later, Gattone et al. demonstrated an impressive decrease in cyst fluid accumulation *via* blockade of the vasopressin V_2_ receptor in rat and mouse models of polycystic kidney disease (38, 39). Subsequently, the multicenter international TEMPO 3:4 study included nearly 1,500 adults (aged 18–50 years) with ADPKD, eGFR above 60 mL/min/1.73m^2^, and relatively large kidneys (TKV > 750 mL) who were randomized to vasopressin V_2_ receptor blockade with tolvaptan or to placebo for 3 years (40, 41). All participants were advised to avoid thirst by drinking a glass of water after each urination. Participants in the tolvaptan arm demonstrated slower annual increase in TKV than controls (2.8 vs. 5.5%; *P* < 0.001) and slower annual decline in kidney function (−2.6 vs. −3.8 mg/mL; *P* < 0.001). Suppression of plasma arginine vasopressin (AVP) concentration was observed in both groups, likely due to voluntary water intake by placebo participants. Thus the observed magnitude of effect between treatment and placebo groups may have been diminished. It is important to note that the TEMPO clinical trial included participants with relatively advanced disease (TKV > 750 mL) and thus the potential benefit to the general ADPKD population may be overestimated based on trial results. It is further unknown if the effect on TKV and renal functional decline would be sustained with ongoing treatment beyond a 3-year period.

Based on these clinical trial results, tolvaptan was subsequently approved for rapidly progressive ADPKD in adults in Europe, Japan, Korea, and Canada but not in the United States. There is nevertheless some variability regarding the definition of “rapid progression” and thus which patients merit therapy. Although it has been proposed that tolvaptan could decrease annualized TKV growth by 1.99% per year (*P* < 0.001) and eGFR decline by 0.40 (*P* = 0.23) in CKD stage 1 (42), for example, European guidelines have recommended against starting tolvaptan in patients aged 30–40 years with CKD stage 1 (eGFR > 90 mL/min/1.73 m^2^) due to slow progression of disease (43). The U.S. Food & Drug Administration denied the new drug application for tolvaptan for ADPKD in 2013, citing concern for side effects (including significant liver enzyme elevation in 4.4%) and high study dropout rate (23 vs. 14%) in the TEMPO trial. Of note, two participants met definition for Hy’s Law case although all cases resolved with drug withdrawal. Despite the positive results of the TEMPO trial, concern also remains regarding the cost-effectiveness of tolvaptan for ADPKD, with a recent Markov-based analysis suggesting that while tolvaptan therapy could prolong median age at end-stage kidney disease by 6.5 years, this came at a cost of $744,100 per quality-adjusted life-year gained (44).

With the positive results of the TEMPO trial, there is great interest to apply tolvaptan to pediatric ADPKD. Several considerations are needed however in planning such a pediatric trial. The severity of structural kidney disease as studied in the TEMPO trial (TKV > 750 mL) is not as common among the general pediatric ADPKD population. Thus, the effect of therapy may be less impressive than in the TEMPO trial, and there are significant potential side effects as detailed above. Furthermore, the vast majority of children with ADPKD maintain normal renal function (CKD stage 1), and a validated method to identify slow vs. rapid progressors does not exist in the pediatric population. Therefore, patient selection criteria must be carefully delineated. Finally, the lower age limit at which children can be expected to appropriately compensate for vaptan-induced polyuria has not been well established. These and other concerns have led to delay in initiation of a pediatric ADPKD clinical trial with tolvaptan although future carefully designed studies should be forthcoming.

It has been proposed that modulation of kidney cyst growth similar to V_2_ receptor blockade might be achieved easily and safely with suppression of AVP *via* chronic high fluid intake (45). Such an intervention is attractive for use in the pediatric age group due to its low risk of potential side effects. The feasibility of inducing low urine osmolality has been demonstrated in a small pilot study in ADPKD adults (46). Thirty-four participants ages 18–60 years with ADPKD and eGFR ≥ 60 mL/min/1.73 m^2^ were randomly assigned to a low-osmolar diet vs. no intervention for a 2-week period. The intervention group demonstrated a significant decrease in urine osmolality and mean plasma copeptin levels, a marker of AVP effect, over the study period. However, a small non-randomized clinical trial of high fluid intake for 1 year in adults with ADPKD was paradoxically associated with increased TKV (47). It remains to be determined whether constant high fluid intake on a prolonged basis is truly practicable in either adults or children, the latter whom may have some difficulty consistently following a low-osmolar diet with protein needs for growth, and thereafter what effect these interventions might have on kidney cyst growth long term.

Somatostatin analogs such as octreotide and lanreotide have been utilized in adults with ADPKD to impact both polycystic liver and polycystic kidney disease. These medications inhibit cAMP production through stimulation of Gαi. The ALADIN study demonstrated diminished growth in TKV after 1 year of treatment with octreotide but the effect was not sustained after 3 years (48, 49). An observational study of 6-month treatment with lanreotide in adults with polycystic liver/kidney disease demonstrated a slight but statistically significant decrease in median TKV (1,023–1,012 mL; *P* = 0.006) (50). Further study is needed to determine whether there is a sustained clinically significant effect of these medications on TKV in ADPKD. Such medications may have a more important role in management of PKD-associated polycystic liver disease, which would be an exceedingly rare occurrence in childhood ADPKD.

Inhibitors of the mammalian target of rapamycin (mTOR) have been previously studied in adult ADPKD. The mTOR pathway plays an important role in cell growth and proliferation, including interaction of the cytoplasmic tail of polycystin 1 with tuberin. Inappropriate activation has been observed in some cyst-lining epithelial cells in human ADPKD patients and in mouse models of ADPKD (51, 52). Animal studies suggested that rapamycin, an inhibitor of mTOR, reduced cystogenesis, an effect which is dependent on appropriate blood levels and the tubular origin of cyst development (52, 53). Such studies led to subsequent clinical trials of mTOR inhibition in adult ADPKD with disappointing results. Sirolimus treatment had no effect on TKV or kidney function over an 18-month period in adults with ADPKD and normal kidney function (54). Gastrointestinal side effects of sirolimus were common, with 82% of treated participants complaining of mucositis. Everolimus treatment for 2 years in adults with ADPKD resulted in a non-significant slower rate of increase in TKV and faster decline in eGFR (55). Cytopenias and mucositis occurred significantly more frequently in the treatment group. These medications have not been studied in pediatric ADPKD, but their use is likely to be limited by the high rate of side effects and concern for possible deleterious effects of long-term immunosuppression beginning early in life.

An alternative approach for suppressing mTOR activity is to utilize agents that activate AMP-activated kinase (AMPK). This kinase has been shown to antagonize mTOR activation (56). In this regard, metformin, a pharmacological activator of AMPK, has been demonstrated to arrest cyst growth in mouse models of PKD (57). Metformin has the advantage of being extensively utilized in clinical and research settings in a variety of medical conditions including diabetes and polycystic ovary syndrome in both adults and children. Thus, ample safety data are available for review. The medication is also relatively inexpensive and may have anti-inflammatory and anti-fibrotic effects [reviewed in Ref. (58)], which could be of potential benefit for vascular health in ADPKD. Clinical trials are currently ongoing to assess the effect of metformin on kidney cyst growth and function in adults with ADPKD (NCT02903511, NCT02656017). With increasing experience with metformin in children with various medical conditions, metformin represents a candidate intervention for children with ADPKD which requires further reflection.

Curcumin, from the plant *curcuma longa*, is a naturally occurring polyphenol found in the spice turmeric, which has the unique ability to activate transcription of key antioxidants, suppress inflammation, and reduce cell proliferation, leading to recent application in conditions of abnormal cell growth including cancer. In various animal models of acute and chronic kidney injury, curcumin treatment has been shown to mitigate renal injury (59–61), possibly by reduced activation of the pro-inflammatory transcription factor NFκB and its downstream target tumor necrosis factor-α (TNF-α), concomitant with an increase in the anti-inflammatory transcription factor peroxisome proliferator-activated receptor-γ (PPARγ) (62, 63). Curcumin has been shown to slow cyst growth *in vitro* in a dose-response manner, using both the Madin–Darby canine kidney cell cyst model and an embryonic kidney cyst model (64). These effects appear at least in part to be mediated by alteration in intracellular signaling proteins like Ras, B-raf, p-MEK, p-ERK, c-fos, and Egr-1. In the Pkd-1 deletion mouse model, curcumin improves renal histology and reduces proliferative index, cystic index, and kidney weight normalized to total body weight (65). Curcumin has also been shown to reduce vascular dysfunction in rodent models of hypertension, diabetes, and aging (66–69). These improvements are associated with increased vascular nitric oxide bioavailability and arterial eNOS expression, reduced vascular oxidative stress (decreased reactive oxygen species, reduced oxidative damage, and increased antioxidant enzymes), and increased activity of hemoxygenase-1, which can promote anti-inflammatory pathways (66–69). Vascular dysfunction is known to occur in adults with ADPKD (70), and cardiovascular disease is the leading cause of mortality in ADPKD (71). Recent studies have shown that children and young adults with ADPKD also have vascular dysfunction as evidenced by impaired endothelial dependent dilation and increased arterial stiffness (72) as compared to healthy children. The results of such studies formed the rationale for a randomized placebo-controlled interventional trial of curcumin therapy in children and young adults with ADPKD (NCT02494141), which is currently ongoing. This study will assess brachial artery flow-mediated dilation and aortic pulse wave velocity at baseline and following 12 months of curcumin vs. placebo. Secondary outcome measures include height-corrected TKV as well as various urine and plasma markers of inflammation and oxidative stress.

## Conclusion

Autosomal dominant polycystic kidney disease is a common kidney condition with major implications for long-term health. Structural kidney disease and vascular dysfunction are evident in childhood, and the earlier we can mitigate these processes, the more favorable the long-term renal and cardiovascular prognoses. It is critical to establish a reliable means to identify children at highest risk for rapid progression and to design safe interventional trials which can be initiated in childhood. Several previously investigated and potential therapeutic interventions have been reviewed here and their relevance to the pediatric ADPKD population discussed. Recent clinical trials suggest benefits of aggressive control of blood pressure with RAAS blockade as well as statin therapy in affected children. Tolvaptan shows great promise to mitigate structural and functional kidney disease progression in adults with advanced ADPKD, but cautious study is needed in affected children. The mechanisms of cyst formation are quite complex. Thus, it seems likely that a combination of treatments will be necessary to optimally inhibit cyst growth and that the ideal combination of therapies may vary over time relative to disease stage in both children and adults.

## Author Contribution

The author confirms being the sole contributor of this work and approved it for publication.

## Conflict of Interest Statement

The author declares that the research was conducted in the absence of any commercial or financial relationships that could be construed as a potential conflict of interest.
